# A Probabilistic Target Search Algorithm Based on Hierarchical Collaboration for Improving Rapidity of Drones

**DOI:** 10.3390/s18082535

**Published:** 2018-08-02

**Authors:** Il-Kyu Ha, You-Ze Cho

**Affiliations:** 1 Department of Computer Engineering, Kyungil University, Gyeongsan 38428, Korea; ikha@kiu.kr; 2 School of Electronics Engineering, Kyungpook National University, Daegu 41566, Korea

**Keywords:** hierarchical search, unmanned aerial vehicle search, drone target detection, hierarchical collaboration search, drone search

## Abstract

Finding a target quickly is one of the most important tasks in drone operations. In particular, rapid target detection is a critical issue for tasks such as finding rescue victims during the golden period, environmental monitoring, locating military facilities, and monitoring natural disasters. Therefore, in this study, an improved hierarchical probabilistic target search algorithm based on the collaboration of drones at different altitudes is proposed. This is a method for reducing the search time and search distance by improving the information transfer methods between high-altitude and low-altitude drones. Specifically, to improve the speed of target detection, a high-altitude drone first performs a search of a wide area. Then, when the probability of existence of the target is higher than a certain threshold, the search information is transmitted to a low-altitude drone which then performs a more detailed search in the identified area. This method takes full advantage of fast searching capabilities at high altitudes. In other words, it reduces the total time and travel distance required for searching by quickly searching a wide search area. Several drone collaboration scenarios that can be performed by two drones at different altitudes are described and compared to the proposed algorithm. Through simulations, the performances of the proposed algorithm and the cooperation scenarios are analyzed. It is demonstrated that methods utilizing hierarchical searches with drones are comparatively excellent and that the proposed algorithm is approximately 13% more effective than a previous method and much better compared to other scenarios.

## 1. Introduction

Rapidity and accuracy are crucial factors for a drone operating in a wide navigation area to find its target in various applications, such as searching for emergency patients who require immediate treatment or the monitoring of natural disasters requiring prompt warnings and responses [1,2]. The rapidity of a drone is particularly important in several applications, such as the rescue of victims within the golden period, monitoring of military facilities and the movement of weapons during wartime, and monitoring of fast-moving natural disasters such as forest fires. Drones spend a significant amount of time searching for targets because they must track exact target locations in a wide range of scenarios. Therefore, there is a need for a technique that enables drones to quickly identify the location of a target within a wide navigation area. Another important factor is accuracy. The quality of information that a drone acquires from a target when executing a mission is affected by various factors, such as the search environment, search altitude, and camera sensor performance, with search altitude being one of the most important factors affecting accuracy. At high altitudes, a drone can cover a wide range, but the quality of the data acquired declines. However, at low altitudes, a drone can obtain high-quality data by searching within a narrow range. Therefore, it is necessary to determine the optimum altitude by considering the performance of a drone’s camera to secure highly accurate data.

In the actual operation of drones, the issue of rapidity is closely related to effective search algorithms, including the establishment of effective search paths, collaboration among drones, and other factors. As mentioned above, the problem of accuracy is often related to drone altitude changes.

This study focused on the improvement of the promptness and accuracy in drone target searching. In particular, it has been determined that the most important factor for improving accuracy in previous studies was search altitude [3]. The aim of this study was to improve the accuracy of target searching by utilizing several search drones to divide the search altitude into different layers. We examined how to exchange search information between drones using the optimum search path.

Various drone target search methods have focused on improving speed. If the task for a drone is urgent, it is necessary to select a search path by judging the information learned by the drone. In this case, a commonly used method is the probabilistic search method [4]. This is a method for determining whether or not a target exists based on a stochastic approach and performing search actions based on initial navigational information.

Therefore, in this study, an improved hierarchical probabilistic target search algorithm based on the collaboration of drones at different altitudes is proposed. In particular, a method to reduce search times and search distances by improving the information transfer between high-altitude and low-altitude drones is proposed. Through simulations, the effectiveness of the proposed method is demonstrated by comparing it to various types of search scenarios. The contributions of this study can be summarized as follows:

- First, an improved hierarchical probabilistic target search algorithm based on the collaboration of drones at different altitudes is proposed. This is a method for reducing the search time and search distance by improving the information transfer methods between high-altitude and low-altitude drones. Specifically, to improve the speed of target detection, a high-altitude drone performs a preliminary search of a wide area.
- Second, this study suggests a method of using thresholds for information transfer between high altitude and low altitude to improve the efficiency of a search, i.e., to reduce the search time and search travel distance. In this method, when the probability of the existence of a target at a high altitude is higher than a certain threshold, the search information is transmitted to a low-altitude drone.
- Third, several drone collaboration scenarios that can be performed by two drones at different altitudes are introduced and compared to the proposed algorithm. These methods are hierarchical cooperation methods of drones that can be used in an actual search. Through simulations, it is demonstrated that methods utilizing hierarchical searches with drones are comparatively excellent and that the proposed algorithm has better performance compared to other scenarios.

In Section 2, target detection methods and altitude control methods are examined, and a target detection method based on a probabilistic search is described. Section 3 describes various improvements for the probabilistic search method and proposes an algorithm that can effectively reduce search distances and times. Section 4 demonstrates the effectiveness of the proposed method by comparing it to various search scenarios. Finally, this paper is concluded in Section 5.

## 2. Related Works

In this section, methods for target searching and control methods for search altitude, which are the most important elements in the target searching of drones, are investigated. A probabilistic search method for autonomous driving is also investigated.

### 2.1. Target Detection Method

Recently, various studies have focused on target searching utilizing unmanned aerial vehicles (UAVs), such as drones. There are three main types of target searching methods in these studies: image-processing-based target searching methods, signal-based target searching methods, and probabilistic target searching methods. Image-processing-based target searching methods aim to determine if a target exists based on images acquired by a UAV camera sensor. It is not a trivial task for the UAV itself to analyze acquired image information and determine if a target exists. Therefore, the acquired images are transmitted to a remote base station for analysis. Signal-based target search methods aim to detect the presence of a target by analyzing signals generated by targets. Finally, probabilistic target search methods aim to identify the existence of a target utilizing only a UAV in a search area with uncertainty regarding the existence of a target.

Liu et al. [5], Wang et al. [6], and Mejias et al. [7] focused on image-processing-based target search methods, with that of Minaeian et al. [8] being the most-recent study. In image-processing-based target search methods, a UAV performs the task of acquiring images of a target utilizing a camera sensor and storage device. The obtained information is transferred to a base station processor and analyzed by various algorithms. Such methods have a major limitation in that it is difficult to quickly identify a target because the acquisition of information, execution of the operation, processing of information, and operational commands are performed by separate computers.

According to Arora et al. [9], magnetic, radar, thermal, acoustic, chemical, electrical, seismic, optical, and ultrasonic signals are generated by various targets. Signal-based target search methods mainly utilize electrical signals. Arora et al. [9], Costa et al. [10], and Jawhar et al. [11] focused on signal-based target search methods. Such methods identify a target by sensing signals generated by that target. In recent years, the applications of UAVs have been expanding and many unspecified targets have often been found. These targets often do not generate any signals. Therefore, these methods are mainly utilized to detect periodic signals sent by a target in a wireless sensor network or to find a target in conjunction with a movement detector on the ground.

To search effectively, it is necessary to predetermine if a target exists in a search area for both image-processing-based target search methods and signal-based target search methods. The criteria for judgment in such methods are the observed images or received signals. In contrast, probabilistic target search methods increase the probability of finding a target by determining the presence or absence of a target in an uncertain search area based on search information.

Probabilistic target search methods can be utilized to identify sequential target existence information for autonomous UAV searches. In such methods, a UAV can directly acquire information regarding a target and determine if a target exists based on this information.

If there is already defined information, such as GPS information, the UAV can determine its action based on that information. However, in the probabilistic search method, without confirmed information, the UAV requires a strategy for selecting the next cell to move to within the search area. Chung et al. [12] categorized strategies for selecting such cells into non-adaptive search strategies and adaptive search strategies. In addition, they defined random walk searches and sweeping searches as non-adaptive search strategies and myopic searches, drosophila-inspired searches, and saccadic searches as adaptive search strategies.

The random walk search method [13] is a representative non-adaptive search strategy that determines movement by randomly selecting one of the current search cells and neighboring search cells. The sweeping search strategy [14] can be applied when a search area is relatively simple and exhibits little change. This strategy is a method of searching each cell sequentially in a vertical or horizontal direction throughout the search area. The myopic search strategy, which is an adaptive search strategy, calculates a belief value for target existence in neighboring cells in movable directions and selects the cell with the highest value. The drosophila-inspired search strategy [15] is a method of selecting the cell with the shortest linear travel distance. The saccadic search strategy [16] selects the cell with the highest confidence level for target existence in a search area.

According to Waharte et al. [17], search algorithms can also be classified into three types: greedy heuristics algorithms, potential-based algorithms, and partially observable Markov decision process (POMDP) algorithms. Greedy heuristics algorithms are a method of selecting the neighboring cell that has the highest probability of target existence based on the target state probability density function of the search area [18,19,20]. Potential-based algorithms find the sum of attractive potential values (values increase as the search moves toward the target) and repulsive potential values (values increase as the search moves closer to an obstacle) of a cell in a search area, then move toward the cell with the minimum potential value [21,22]. POMDP algorithms represent a method of performing sequential actions to obtain an expected value when the current action is determined after obtaining the value of the current state by navigating a regional portion of a search area [4,23].

### 2.2. Target Detection Based on Probabilistic Search

In the previous section, it was stated that a probabilistic target search method is a method of determining whether or not a target exists based on stochastic evaluation while performing search actions based on initial navigational information. Such methods can be utilized to determine sequential target existence for autonomous searching by UAVs.

Several studies on probabilistic target search methods are discussed below. Chung et al. [4] defined a probabilistic target search problem for drones, proposed a search model based on false detection and error detection, and proposed a cyclic probability update function that sequentially updated the probability of target existence within a search cell. This study was followed by Chung et al. [12] which refined both the search problem and probability update function. This study also analyzed a search strategy for selecting the next cell to move to within a search area.

Studies were performed by Waharte et al. [17,20,23], Symington et al. [18], and Morse et al. [19]. Waharte et al. [23] defined the search problem utilizing mathematical notation and experimented with schemes utilizing collaboration between two drones. In [20], the authors studied a case where the search range of the drone spreads between the current search cell and neighboring cells, and proposed a search algorithm considering search altitude. Waharte et al. [17] compared the performances of several conventional search algorithms, including greedy heuristics, potential-based heuristics, and POMDP-based heuristics, through various simulations. They also examined the cooperation of drones at different altitudes. Finally, Symington et al. [18] presented a function that updated the probability of target existence in a search cell iteratively during search execution in a probabilistic search method.

For probabilistic searches, each drone has a probabilistic map (belief map or initial map) composed of |A| cells. Each cell in the belief map has a probability that a target exists in that cell. This probability is roughly predicted utilizing a Gaussian distribution [24] or based on environmental features such as rivers or roads. Based on the belief map, after each observation, the probability distribution function of a target state is recomputed utilizing Equation (1). This equation was derived via Bayesian filtering [25], and the observed value of a drone influences the probability function [4,12]:

(1) $$\mathrm{Pr}\left(x_{T}=a|D^{t}\right)=\frac{\mathrm{Pr}(d_{a}^{t}|x_{T}=a,\;D^{t-1})}{\mathrm{Pr}(d_{a}^{t}|D^{t-1})}\mathrm{Pr}\left(x_{T}=a|D^{t-1}\right).$$

In Equation (1), $d_{a}^{t}$ is the observation of a drone in cell ‘*a*’ at time *t*, *D^t^* becomes a set of observations up to time t as {*d*^1^, …, *d^t^*}, and *x_T_* = *a* indicates that a target exists in cell a. Therefore, Pr(*x_T_* = *a*|*D^t^*) is the probability of a target being present in cell ‘*a*’ for the time step *t*. Pr(*x_T_* = *a*|*D*^*t*−1^) is the probability for the previous time step. Pr($d_{a}^{t}$|*D^t^*^−1^) is the marginalization of the measurement as Equation (2). In Equation (2), H is a binary variable indicating whether a target exists in the search area. Pr($d_{a}^{t}$|*x_T_* = *a*, *D^t^*) is the search result of cell ‘*a*’ at time t and is obtained from the search model of Equation (3):

(2) $$\mathrm{Pr}\left(d_{a}^{t}|D^{t-1}\right)=\left[\begin{array}{c} \mathrm{Pr}\left(d_{a}^{t}|x_{T}=a,\;D^{t-1}\right)\mathrm{Pr}\left(x_{T}=a|D^{t-1}\right)\\ +Pr(d_{a}^{t}|H=0,D^{t-1})\left(1-Pr\left(x_{T}=a|D^{t-1}\right)\right) \end{array}\right].$$

During the iterative calculation, if the value of Pr is greater than a threshold probability value, it can be regarded as a detection success.

In the problem of the probability search, the following detection model [4,12,18] is considered:

(3) $$\mathrm{Pr}\left(d_{a}^{t}|x_{T}\right):\;\{\begin{array}{c} \mathrm{Pr}\left(d_{a}^{t}=0|x_{T}=a\right)=\beta ,\\ \mathrm{Pr}\left(d_{a}^{t}=1|x_{T}=a\right)=1-\beta ,\\ \mathrm{Pr}\left(d_{a}^{t}=0|x_{T}\neq a\right)=1-\alpha ,\\ \mathrm{Pr}\left(d_{a}^{t}=1|x_{T}\neq a\right)=\alpha . \end{array}$$

In Equation (3), $d_{a}^{t}$ is the observed value measured at time t for cell ‘*a*’, *x_T_* indicates whether the actual target exists in cell *a*, *α* is the false alarm probability, and *β* is the missed detection probability. That is, *α* denotes that the target does not actually exist in cell ‘*a*’ but the observed value denotes that there is *a* target, and *β* denotes the case where the target actually exists in cell ‘*a*’ but the observed value denotes that there is no target.

Therefore, Equation (4) is derived from Equation (1) [18]. The updated equation for the recursive Bayesian estimator [21,26] is defined below based on whether the search case is positive or negative:

(4) $$P_{t}=\{\begin{array}{c} \frac{\left(1-\beta_{h}\right)P_{t-1}}{\left(1-\beta_{h}\right)P_{t-1}+\alpha_{h}\left(1-P_{t-1}\right)},\;if\;d^{t}=1\\ \frac{\left(\beta_{h}\right)P_{t-1}}{\left(\beta_{h}\right)P_{t-1}+\left(1-\alpha_{h}\right)\left(1-P_{t-1}\right)},\;if\;d^{t}=0 \end{array}.$$

According to Equation (4), if the value of *d^t^* is one, then the probability value of the cell is calculated utilizing the upper expression in Equation (4). If the value of *d^t^* is zero, the lower expression is utilized.

Symington et al. [18] analyzed the experimental determination of the values of *α* and *β* in the probability update function for a probabilistic search, where *α* is the probability of false alarms and *β* is the probability of missed detection.

### 2.3. Altitude Control Strategies

The search altitude of drones is a very important factor in real-world operations because changes in the search altitude significantly affect the speed and accuracy of the drone. The altitude of a drone in a search strategy utilizing a single drone can be an important factor for improving the performance of a search. In a search utilizing a group of drones, the performance of the search can be improved via cooperation of drones performing searches at different altitudes. Studies related to the changes in altitude of drones are discussed below.

Smaragdis et al. [25] defined a navigation problem and proposed a navigation model based on probabilistic methods. This model is significant as a starting point for tackling accuracy issues related to altitude. Waharte et al. [17] introduced algorithms related to the altitude of drones and simulated drone performance in a specific experimental environment and performed comparative analysis of the results. Waharte et al. [23] presented an early study that focused on a cooperation method between a high-altitude drone and a low-altitude drone. However, this study did not present specific navigation algorithms and focused mainly on a comparison between the cooperation and non-cooperation of two drones at different altitudes. Kim et al. [27] compared the navigational performance of two drones at different altitudes utilizing the POMDP method. Another study based on probabilistic acquisition methods presented a study on the changes in the maximum probability of search success by drones at varying altitudes [22].

## 3. Advanced Hierarchical Probabilistic Search Algorithm

In this section, altitude control strategies for drone target detection are considered and an improved hierarchical probabilistic search algorithm is proposed.

### 3.1. Improvement of the Probabilistic Search

Navigating an entire search space based on a probabilistic search method requires a large amount of time to compute complex probability functions. Therefore, a method is needed that reduces the number of calculations of the probability function and can detect a target within the shortest possible time. Here, the basic concepts of a hierarchical probabilistic search method that will be presented in the next section are described. This method is a search method that operates via cooperation between high-altitude drones and low-altitude drones. It sequentially searches an area corresponding to four times the search range at the height of the high-altitude drone and selects a region for more precise searching, which is then searched by low-level drones. A simplified algorithm representing this concept is presented in Algorithm 1. In the algorithm, Th^b^ is the threshold value for target detection success, Max^b^ is the highest belief value at the current altitude, Area^h^ is the area having the highest belief value, and Th^n^ is the threshold of reliability for transmitting information from a higher drone to a lower drone. Checking this value to change the search altitude is one of the most important aspects of our algorithm.

First, a drone will initially search the search area at a high altitude. It searches four search areas sequentially and computes a probability (belief) value for each cell in the search areas. It then selects the area with the highest belief value and retains the search information for the low-altitude search. If the highest reliability value is above the threshold Th^n^, then the high-altitude drone transmits the search information to the low-level drone. This initial search is performed to obtain the advantages of a high altitude, namely search efficiency as a result of covering a wide search area by a small travel distance and continuously searching at as high an altitude as possible. This is an important part of the proposed algorithm. The low-altitude drone performs a more specific search based on the search information from the high-altitude drone. When the highest probability value is greater than or equal to the threshold value, target searching stops and the target is considered to be found.

**Algorithm 1** Basic Outline of Proposed Algorithm1:**while** Max^b^ < Th^b^2: //high-altitude search3: high-altitude drone searches for the target sequentially in four search areas4: and computes a belief value for each cell in the search areas,5: it then selects an Area^h^6: **if** Max^b^ > Th^n^
**then**
7:  //low-altitude search8:  send search information to the low-altitude drone,9:  low-altitude drone searches for the target sequentially in the Area^h^10:  and computes Max^b^11: **end**12: **end**13: stop searching

### 3.2. Altitude Control Strategy

**Figure 1:**
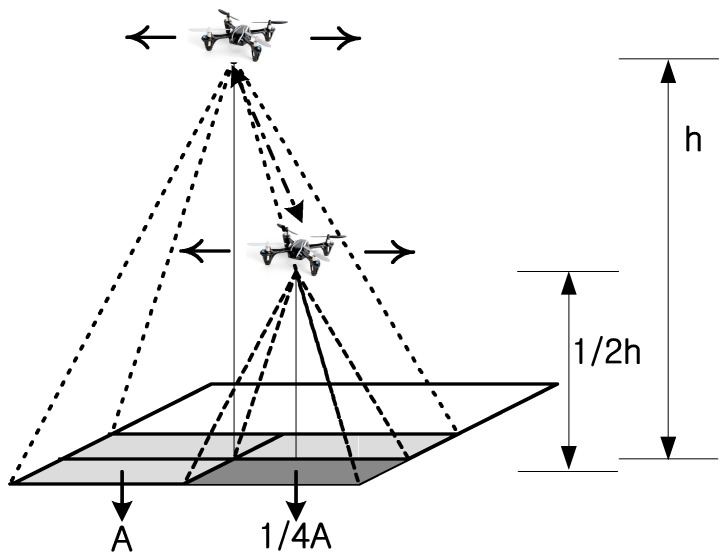
Search area according to search altitude of drone.

The algorithm proposed above is a target search method based on the cooperation of drones at different altitudes. For target detection based on the collaboration of drones at different altitudes, we considered the following two issues. Our first consideration was the division of the altitude (height) of navigation (i.e., how to divide the altitude of navigation into several different levels), which is dependent on the number of drones participating in the detection process. Second, we considered the partitioning of the navigation area (i.e., the partitioning of a search area into clusters of an optimal size). First, the first consideration will be examined. As shown in Figure 1, if the altitude of a drone is h, then the search area of that drone is A. When the altitude of a drone is a half of h, then the search area of that drone is 1/4A. Several observations based on this relationship can be made. Most importantly, the search area is proportional to the square of the altitude.

Next, the shape of the search area is considered. Generally, a drone's camera has a wide-angle lens. Although the acquired images seem to be wide, for the sake of both convenience and accuracy of calculations, the search area should be considered in the form of a rectangle. Assuming the search area is a rectangle, the relationship between the search altitude of a drone and the search area is defined by Equation (5). If we know the search angle *a*’ and altitude of the drone *h*, the search area can be calculated [3]:

(5) $$A=2{\left(\frac{h}{\tan a^{\prime }}\right)}^{2}.$$

### 3.3. Advanced Hierarchical Probabilistic Search Algorithm

An improved hierarchical probabilistic search algorithm that considers the search altitude and search area of the drone is proposed. The key points of the proposed method are summarized below. To improve the speed of target detection, a high-altitude drone first performs a search of a wide area. When the probability of the existence of a target is higher than a certain threshold, the search information is sent to a low-altitude drone, which then performs a more specific search. This method fully exploits the advantages of a high-altitude search. Specifically, it exploits the advantage of reducing the time and travel distance required to search a wide search area.

**Figure 2:**
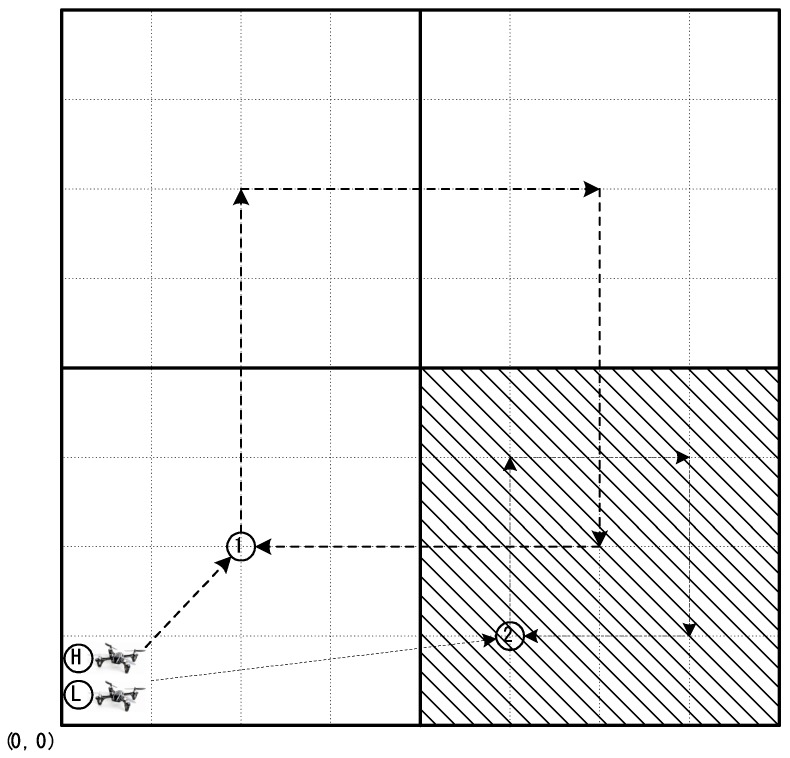
Search process based on collaboration between high-altitude and low-altitude drones.

Figure 2 presents the search process based on the collaboration between a high-altitude and low-altitude drone. The high-altitude drone starts at the origin and sequentially searches areas corresponding to four times its own search area. At this point, it is assumed that the search range of the high-altitude drone is 4 × 4 units and the search range of the low altitude drone is 2 × 2 units. The high-altitude drone sequentially searches all the search regions and then calculates the probability of target existence for each of the cells within each search range. The drone selects the search range with the highest probability of target existence and checks if the probability of target existence is greater than or equal to a predetermined threshold. If the probability of the presence of the target in a selected search range such as the shaded area in Figure 2 is greater than or equal to the threshold, then the high-altitude drone sends the search information to the low-altitude drone. The low altitude drone then performs a more detailed search in this search area based on the received information.

As shown in Figure 2, the drones begin searching from the origin (position (0, 0)) at a high altitude. At this time, the altitude of the first drone is lower than the maximum height limit. It first moves to position 1 in Figure 2. It then searches four high-altitude search areas sequentially and identifies the high-altitude search area containing the cell with the highest target existence probability. If the highest probability is larger than the predefined threshold, the high-altitude drone sends search information to the low altitude drone. Otherwise, the high altitude drone performs high-altitude searches until the highest probability value reaches the threshold. When the highest probability value exceeds the search success threshold, the drones can terminate the search task because the high probability level can be regarded as a successful target detection. When arriving at location 2, the low-altitude drone performs a detailed search at a low altitude. Similar to the search task at a high altitude, this drone sequentially searches four low-altitude search areas and identifies the low-altitude search area containing the cell with the highest target existence probability. If the highest probability is larger than the threshold, the drone can terminate its search task because the probability can be regarded as a successful target detection. If the search probability does not reach the search success threshold value, the search begins again at a high altitude.

If the search probability does not reach the threshold value after searches at high and low altitudes have been repeated more than a certain number of times, the drone moves to the neighboring area and continues searching. At this point, the movement direction can be up, down, left, or right according to the defined selection method (e.g., random search, sweeping search, or saccadic search [4]).

Algorithm 2 presents the process of the proposed hierarchical search algorithm. The algorithm is divided into an initialization phase, high-altitude search phase, and low-altitude search phase. To proceed to the next step in the search process, two threshold values, namely a threshold value Th^sp^ for search success and threshold value Th^lp^ for low-altitude movement, are utilized. The main goal of this algorithm is to reduce the time spent searching at low altitude as much as possible and to increase the probability of finding a target by searching at a high altitude utilizing the conditional query in line 16 of the algorithm. Table 1 lists the definitions of the variables utilized in the proposed algorithm.

**Algorithm 2** Downward Delay Search1: //Initialize2: **found** = 0;3: **round_h_** = 0;4: **round_l_** = 0; 5: **while found** ! = 16: *//High-altitude search*7: **if round^h^** < round_limit **then**8:  *the drone searches four*
**Area^h^**s *at high altitude*9:  *select the*
**Area^h^**
*containing the cell with the highest probability*10:  **if HP^h^** ≥ **Th^sp^ then**11:   **found** = 112:   *stop searching*13:   *break*14:  **else**15:   **if HP^h^** > **Th^lp^ then**16:    *//Low-altitude search*17:    **if round^l^** < round_limit **then**18:     *send information to low-altitude drone*19:     *low-altitude drone searches four*
**Area^l^**s *at low altitude*20:     **if HP^l^** ≥ **Th^sp^ then**21:      **found** = 1 22:      *break*23:     **end**24:    **else**25:     *change altitude upward*26:    **end**27:   **end**28:  **end**29: **else**30:  *move to the next area for another high-altitude search*31: **end**32: **round^h^** = **round^h^** + 133: **end**

## 4. Simulation

In this section, several collaboration scenarios performed by two drones at different altitudes are described. We then compare these scenarios to the proposed algorithm. Through various simulations, we analyze the performance of the proposed algorithm and other cooperation scenarios.

### 4.1. Simulation Environment

**Figure 3:**
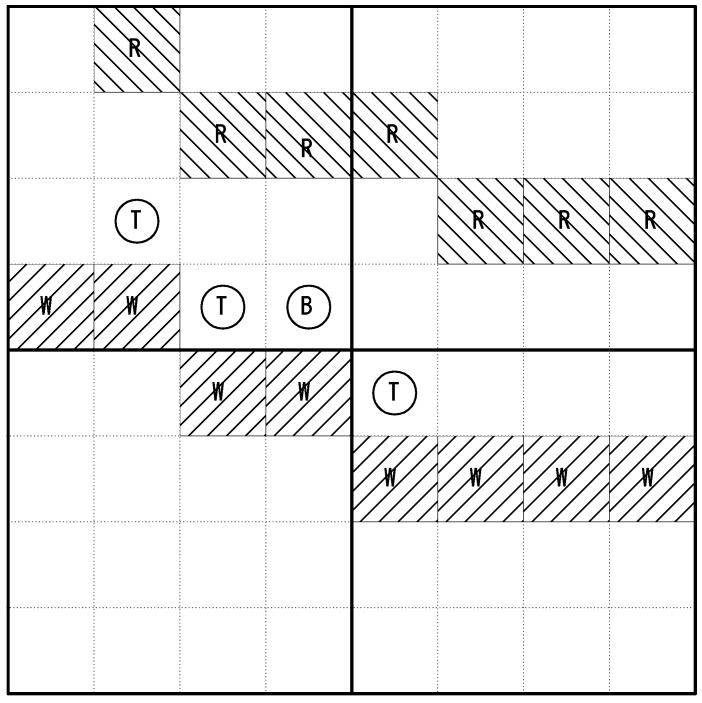
Search map for simulation.

For our simulations, the 8 × 8 unit search map shown in Figure 3 was utilized. In this search map, it was assumed that landmarks such as rivers (R), trails (W), trees (T), buildings (B) exist and only one target exists at a fixed location. The targets considered in this study were static targets on the ground. Though not specified in the text, an example is a person who has lost consciousness and is lying on the ground. Operating on the assumption that the drone knows the information regarding the landmarks on the map based on a rough initial search, the weight values necessary for calculating prior belief according to the probability of target existence in each cell is provided. A value of +0.2 weight to a trail is applied because the target is likely to be in that area. In addition, a value of −0.2 weight for a river, −0.2 for a building, −0.1 for a tree, and +0.25 for an object similar to the target is applied. The weight of a cell with no landmarks is set to zero. It was assumed that the target was located at a random point within the search area.

The specific environment for simulation is described in Table 2. The search area of the high-altitude drone was 4 × 4 units, and the search area of the low-altitude drone was 2 × 2 units. The initial probability of all cells started at 0.5. The initial belief was based on a prior probability distribution function. It was assumed that the drone knew the terrain information of the search area based on a rough search. Therefore, the initial probability of each cell was determined according to the objects in the search area.

A drone regarded a search as yielding a positive detection when the probability value after observing a cell is 0.5 or more and regarded a search as yielding a negative detection if it is 0.5 or less. Based on positive detection or negative detection, the probability of the cell is calculated based on Equation (3). The weight value of the initial belief map was then assigned to *P*_*t*−1_.

The high-altitude drone calculates the target existence probability of each cell in each area and then selects the search area with the highest probability of target existence. During the selection of the search area, it is possible to select the search area with the highest average value of all cell probability values or the search area with the single highest cell probability. The low-altitude drone begins its search based on this information. A belief map is then applied in the same manner as above but with different values of *α* and *β*.

The probability of *α* (false detection) and *β* (missed detection) according to the altitude of a drone search can be obtained experimentally [17]. In this simulation, the values of *α* and *β* from the experimental results of Waharte et al. [17] were utilized. Specifically, 0.00130 as *α* and 0.34593 as *β* for the high-altitude drone and 0.06286 as *α* and 0.20000 as *β* for the low-altitude drone were used. All simulations were executed in MATLAB 2015a.

In the initial state, all cells had the same value as an initial probability value. The sum of the probabilities of all cells was one (delta value in Waharte et al. [23]). Then, the belief map was updated based on the weight values of the feature objects. Values ranging from −0.1 to +0.25 were added to the probability values of each cell based on the feature objects contained in each cell. For example, a hiking trail added +0.2, a river −0.2, a tree −0.1, a building −0.2, and an object similar to the target +0.25.

### 4.2. Search Scenarios

In this section, various search scenarios for drones for use in simulations to perform performance analyses are introduced. The first scenario is the initial version of the proposed algorithm. This is a scenario that does not check the time required to transfer control from a high-altitude to a low-altitude drone. The second scenario is a search scenario based on a linear search by low-altitude drones. The third scenario is a search scenario based on a different cooperation method between a high-altitude and low-altitude drone. The fourth scenario is a search scenario based on a linear search by high-altitude drones. The fifth scenario is the full version of the proposed algorithm.

- Scenario 1
  This scenario is the initial version of the proposed algorithm. In this method, the first drone searches four high-altitude search areas for quick navigation. Then, the area with the highest probability is selected and searched more precisely by a drone at a low altitude. In this method, control is transferred from the high-altitude drone to the low-altitude drone without verifying control transfer. This method operates based on a hierarchical control of drones.
- Scenario 2
  In the second scenario, a low-altitude drone performs a linear probability search. Specifically, the drone searches each low-altitude search area linearly. The low-altitude drone moves linearly in the direction in which the values of x and y increase and searches for the target. The values of *α* and *β* of the low-altitude drone are applied to obtain the target existence probability. The low-altitude drone continues searching based on the *α* and *β* values. The probability of the existence of a target in each cell is calculated recursively utilizing Equation (4).
- Scenario 3
  The third scenario utilizes another altitude-control strategy to detect a target in the search area. The search scenario is as follows. Unlike Scenario 1, the drone searches only one high-altitude search area. The drone selects a low-altitude search area (2 × 2) within the high-altitude search area and sends the search information to the low-altitude drone for more precise searching of the low-altitude search area. This drone then searches the low-altitude search area in detail.
- Scenario 4
  In this scenario, a high-altitude drone performs a linear probability search. This scenario is very similar to the second scenario. The only difference is that the drone is at a high altitude. In addition, since it has a higher altitude, different α and β values for high altitude are utilized to calculate the probability of each cell.
- Scenario 5
  This scenario represents the full method proposed in this study. The high-altitude drone sequentially searches an area corresponding to four times its search range from a high altitude. The search range containing the cell with the highest probability of existence of the target is selected. The drone then checks if the highest probability of target existence is greater than or equal to a threshold value to determine if the search control should be transferred to the low-altitude drone. If the value is above the threshold, the search information is transmitted to the low-altitude drone, which then performs a more precise search at a low altitude.

### 4.3. Simulation Results and Analysis

**Figure 4:**
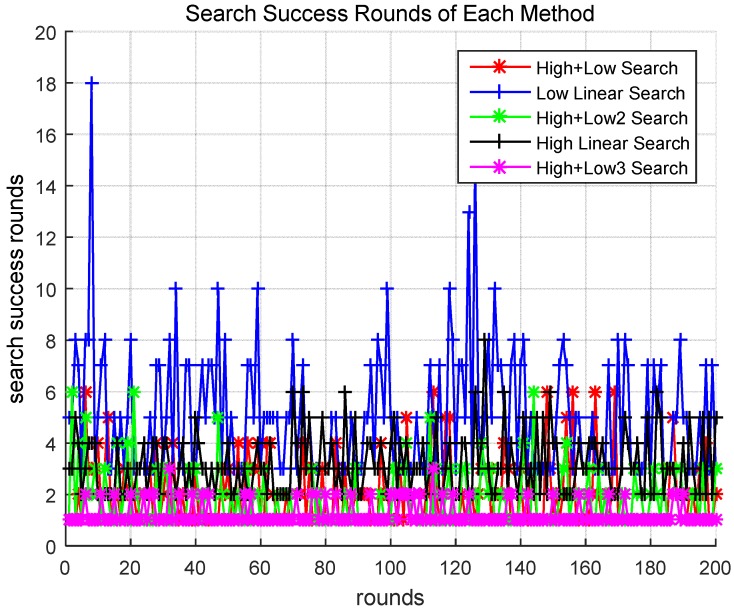
Rounds of target detection required for success of different scenarios.

The above scenarios were compared in our initial simulations. Figure 4 presents comparisons of the number of target detection success rounds of the above scenarios. As shown in the figure, in Scenarios 1 (High + Low), 3 (High + Low2), and 5 (High + Low3), the target was found within seven rounds. However, in Scenarios 2 (Low Linear) and 4 (High Linear), most targets were only found after more than seven rounds. Therefore, one can see that the methods utilizing hierarchical drone searches were relatively more effective. When comparing Scenarios 1 and 5, which both utilize a hierarchical search strategy for the drones, the drones find targets after fewer rounds in Scenario 5. This can be seen more clearly in the following results.

The search success round on the *y*-axis in Figure 4 is the number of times the entire search area was searched until the search succeeded, and the rounds on the *x*-axis is the number of times the simulation was repeated until the search succeeded.

**Figure 5:**
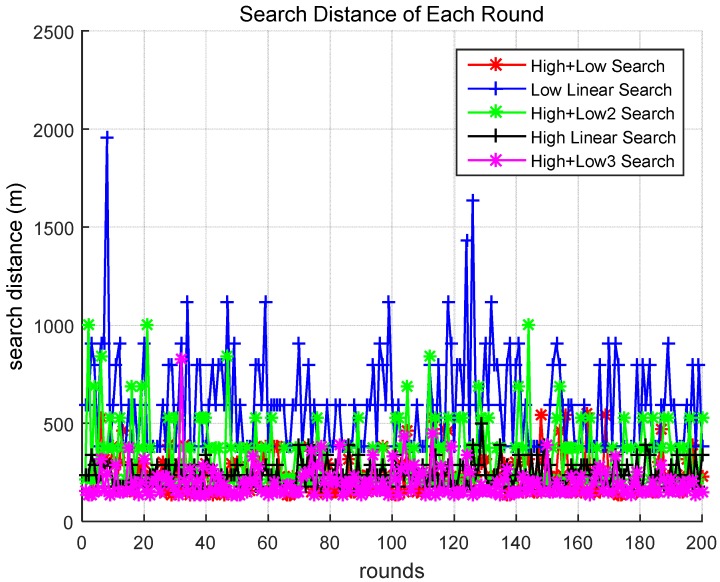
Search distance in each round of different scenarios.

Figure 5 compares the search travel distances of the different scenarios. The search distances were calculated utilizing Equations (5) and (6), and include the number of units moved before the target was found at the altitude of each drone. It was assumed that the height of the high-altitude drone was 20 m and the height of the low-altitude drone was 10 m as shown in Table 2:

(6) $$D=U_{m}\times \sqrt{2\times {\left(\frac{1}{\tan a_{hb}}\times h\right)}^{2}}.$$

In Equation (6), *D* is the search distance, *U_m_* is the number of units moved, tan *a_hb_* is the tangent angle of the hypotenuse and base, and *h* is the altitude height of a drone.

As shown in Figure 5, when the target was found in Scenarios 1 and 5, it was found by moving a smaller distance compared to that of the other scenarios. It was observed that the search method in Scenario 5 moved a smaller distance compared to the method in Scenario 1 before finding the target.

**Figure 6:**
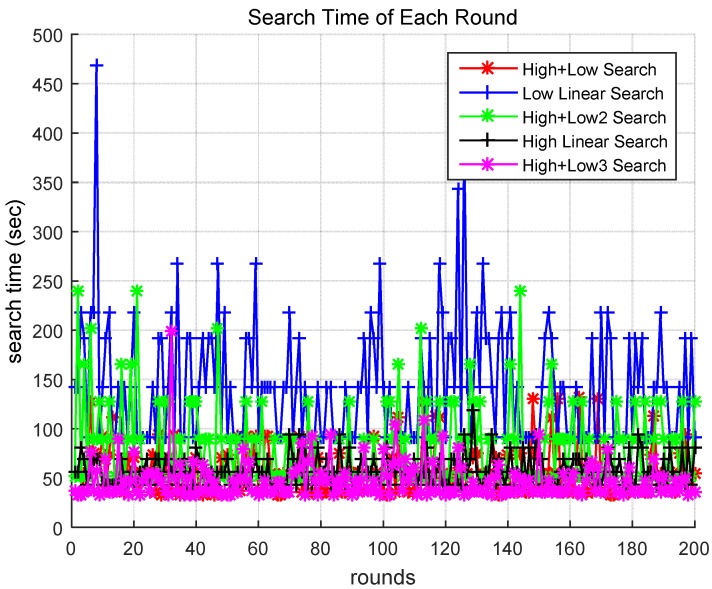
Search time in each round of different scenarios.

Figure 6 compares the search times of the different scenarios. The search time was calculated utilizing Equation (7). We assumed that the drone speed was 15 km/h.

(7) $$T_{s}=S_{d}÷V_{d}.$$

In Equation (7), *T_s_* is the search time, *S_d_* is the search distance, and *V_d_* is the drone velocity. As shown in Figure 6, the drone required a relatively small amount of time to perform the search utilizing the methods of Scenarios 1 and 5.

**Figure 7:**
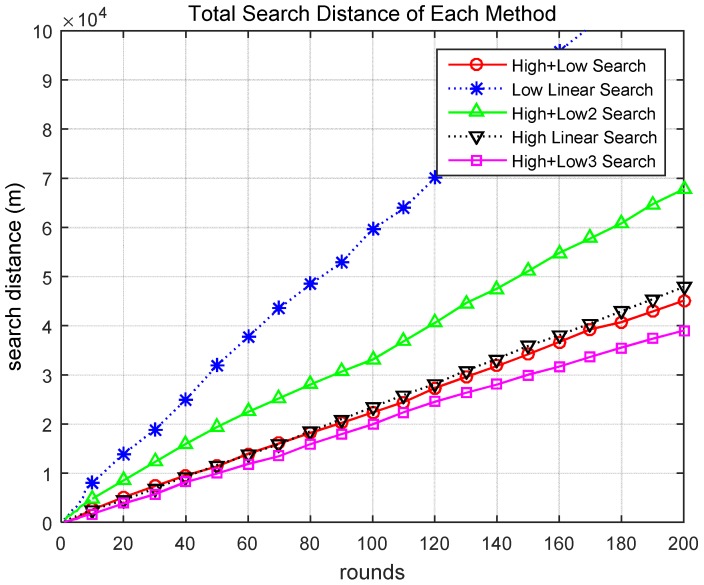
Total search distance of different scenarios.

**Figure 8:**
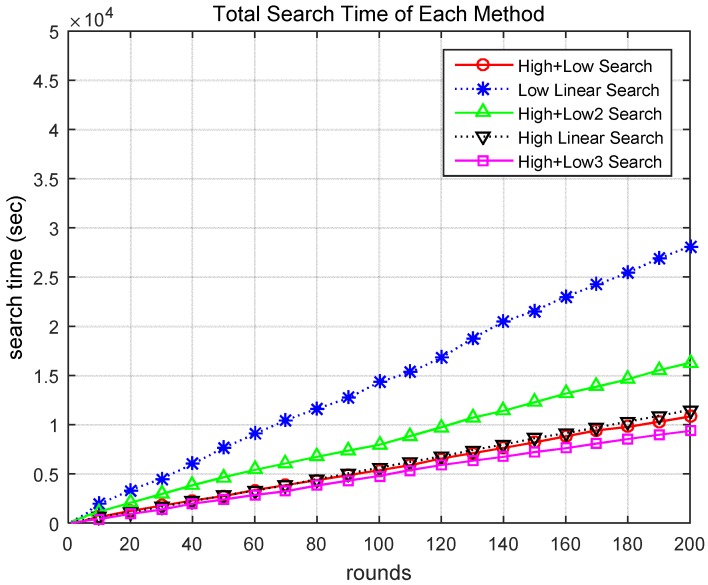
Total search time of different scenarios.

Figure 7 presents the cumulative search distances for the different scenarios. When searching utilizing the methods of Scenarios 1 and 5, the task was performed by moving a relatively small distance. In addition, there is a clear difference between Scenarios 1 and 5. Scenario 5 (pink line) had superior performance compared to Scenario 1 (red line). This difference is more clearly observed in Figure 8.

**Figure 9:**
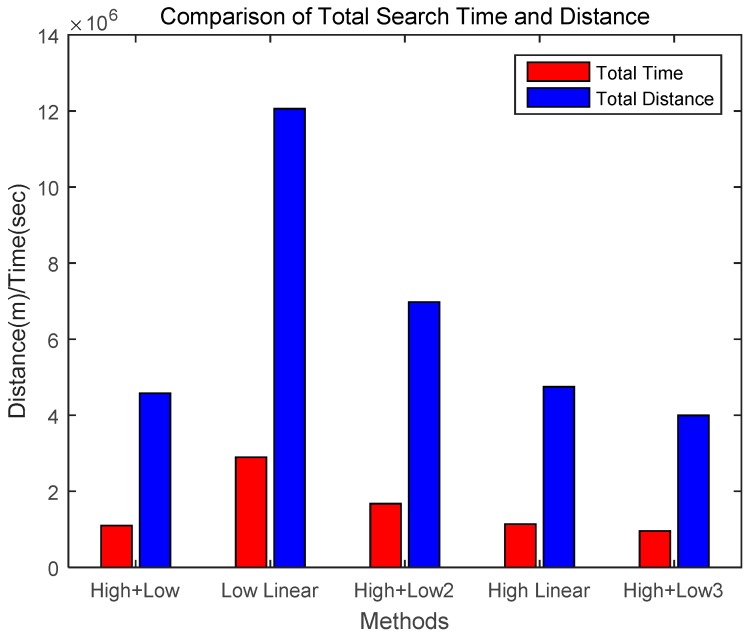
Comparison of total search times and distances of different scenarios.

Figure 8 presents the cumulative search time for the different scenarios. When searching utilizing the methods of Scenarios 1 and 5, the task was performed in a relatively short time. Figure 9 compares the total search time and total search distance over 200 rounds. The proposed Scenario 5 (High + Low3) was approximately 13% more effective than Scenario 1 (High + Low) and showed significantly better performance than the other scenarios. Therefore, through these simulations, it was demonstrated that the proposed method for finding a target based on a hierarchical search by drones is superior to previous methods using general altitude control methods.

Table 3 shows the performance comparison between the proposed method and other scenarios. The ‘Total Time’ and ‘Total Distance’ columns of the table show cumulative search times and search distances of search success when 200 rounds were executed with each method and scenario. The rightmost column shows the High + Low method and the comparative performance of the other methods. As shown, when the total cumulative time and distance for the search success on the “High + Low” method was 100%, the proposed High + Low3 method was 87% of the search success of the High + Low method. Therefore, the proposed method performed better than the other methods.

## 5. Conclusions

In this study, an improved hierarchical probabilistic target search algorithm based on the collaboration of drones at different altitudes was proposed. This method reduced the search time and search distance by improving the information transfer between high-altitude drones and low-altitude drones. The proposed method is basically a search method based on the cooperation of high-altitude and low-altitude drones.

The key points of the proposed method are as follows. To improve the speed of target detection, a high-altitude drone first performs a search of a wide area. When the probability of existence of a target is higher than a certain threshold, the search information is sent to a low-altitude drone. The low-altitude drone then performs a more detailed search in the specified area. This method fully exploits the advantages of searching at a high altitude by reducing the time and travel distance required to search a wide search area.

Through various simulations, the effectiveness of the proposed method was demonstrated. In these simulations, several drone collaboration scenarios performed by two drones at different altitudes were compared to the proposed algorithm. The performance of the proposed algorithm and the different cooperation scenarios was analyzed, and it was found that methods utilizing a hierarchical search by multiple drones are superior to traditional search methods. The proposed method was approximately 13% more effective than previous methods and showed much better performance than the other scenarios. It was proved that the proposed method for finding a target based on a hierarchical search by drones is superior to previous methods using only general altitude-control methods. The contributions and conclusions of this study are summarized as follows:

- This study proposed an improved hierarchical probabilistic target search algorithm based on the collaboration of drones at different altitudes. This method reduced the search time and search travel distance by improving the information transfer between high-altitude and low-altitude drones. In addition, the information transfer method increased the efficiency of the proposed algorithm by using thresholds in the information transmission process.
- This study introduced several drone collaboration scenarios performed by two drones at different altitudes and compared the scenarios to the proposed algorithm. Through simulations, the performance of the proposed algorithm and the cooperation scenarios were analyzed. It was demonstrated that methods utilizing hierarchical searches with drones are comparatively excellent and that the proposed algorithm is approximately 13% more effective than a previous method with much better performance compared to other scenarios.

## Figures and Tables

**Table 1:** Variable definitions.

Variable	Definition
**Area^h^**	search area of drone at high altitude
**Area^l^**	search area of drone at low altitude
**Th^sp^**	threshold probability value for search success
**Th^lp^**	threshold probability value for low-altitude search
**HP^h^**	highest probability among cells at high altitude
**HP^l^**	highest probability among cells at low altitude
**found**	binary variable for found alarm
**round^h^**	number of rounds executed at high altitude
**round^l^**	number of rounds executed at low altitude

**Table 2:** Simulation environment.

Category	Value
Size of search area	8 × 8 units
Number of drones	2
Average drone speed	15 km/h
High-altitude search area of drone	4 × 4 units (altitude: 20 m)
Low-altitude search area of drone	2 × 2 units (altitude: 10 m)
Threshold probability1 (TH^sp^)	0.95
Threshold probability2 (TH^lp^)	0.75
Length of a side of one unit	7.592 m
Probability variables for high-altitude drone	*α* = 0.00130, *β* = 0.34593 [17]
Probability variables for low-altitude drone	*α* = 0.06286, *β* = 0.20000 [17]

**Table 3:** Performance comparison between proposed method and other scenarios.

Algorithm	Search Method	Total Time (sec)	Total Distance (m)	Comparison (%)
**High + Low**	After high-altitude search, low-altitude search	1,099,028	4,579,283	100
**Low Linear**	Search linearly at low altitude	2,893,980	12,058,251	263
**High + Low2**	After high-altitude search, low-altitude search (search by one high-altitude area)	1,673,795	6,974,146	152
**High Linear**	Search linearly at high altitude	1,139,097	4,746,240	104
**High + Low3 (Proposed)**	After high-altitude search with threshold value, low-altitude search	959,327	3,997,199	87
